# Objective and subjective measures of sleep initiation are differentially associated with DNA methylation in adolescents

**DOI:** 10.1186/s13148-023-01553-2

**Published:** 2023-08-26

**Authors:** Michael Larsen, Fan He, Yuka Imamura Kawasawa, Arthur Berg, Alexandros N. Vgontzas, Duanping Liao, Edward O. Bixler, Julio Fernandez-Mendoza

**Affiliations:** 1 Sleep Research and Treatment Center, Department of Psychiatry & Behavioral Health, The Pennsylvania State University College of Medicine, Hershey, PA 17033 USA; 2 Department of Public Health Sciences, The Pennsylvania State University College of Medicine, Hershey, PA 17033 USA; 3 Departments of Biochemistry and Molecular Biology and Pharmacology, Institute for Personalized Medicine, The Pennsylvania State University College of Medicine, Hershey, PA 17033 USA

**Keywords:** Sleep initiation, Epigenetics, DNA methylation, Sleep latency, Bedtime

## Abstract

**Introduction:**

The onset of puberty is associated with a shift in the circadian timing of sleep, leading to delayed sleep initiation [i.e., later sleep onset time (SOT)] due to later bedtimes and/or longer sleep onset latency (SOL). Several genome-wide association studies (GWAS) have identified genes that may be involved in the etiology of sleep phenotypes. However, circadian rhythms are also epigenetically regulated; therefore, epigenetic biomarkers may provide insight into the physiology of the pubertal sleep onset shift and the pathophysiology of prolonged or delayed sleep initiation.

**Results:**

The gene-wide analysis indicated differential methylation within or around 1818 unique genes across the sleep initiation measurements using self-report, actigraphy (ACT), and polysomnography (PSG), while GWAS-informed analysis yielded 67 genes. Gene hits were identified for bedtime (PSG), SOL (subjective, ACT and PSG) and SOT (subjective and PSG). DNA methylation within 12 genes was associated with both subjective and PSG-measured SOL, 31 with both ACT- and PSG-measured SOL, 19 with both subjective and ACT-measured SOL, and one gene (*SMG1P2*) had methylation sites associated with subjective, ACT- and PSG-measured SOL.

**Conclusions:**

Objective and subjective sleep initiation in adolescents is associated with altered DNA methylation in genes previously identified in adult GWAS of sleep and circadian phenotypes. Additionally, our data provide evidence for a potential epigenetic link between habitual (subjective and ACT) SOL and in-lab SOT and DNA methylation in and around genes involved in circadian regulation (i.e., *RASD1*, *RAI1*), cardiometabolic disorders (i.e., *FADS1*, *WNK1*, *SLC5A6*), and neuropsychiatric disorders (i.e., *PRR7*, *SDK1*, *FAM172A*). If validated, these sites may provide valuable targets for early detection and prevention of disorders involving prolonged or delayed SOT, such as insomnia, delayed sleep phase, and their comorbidity.

**Supplementary Information:**

The online version contains supplementary material available at 10.1186/s13148-023-01553-2.

## Background

The onset of puberty is associated with a shift in the circadian timing of sleep leading to delayed sleep initiation, as bedtimes become later or sleep onset latency (SOL) prolonged [1]. This shift toward a later sleep onset time (SOT) is due to maturational changes in the circadian and sleep homeostatic systems as children transition to adolescence [2]. Falling asleep requires circadian, homeostatic, de-arousal and cognitive processes [3], which are highly regulated by the interplay of environmental, psychosocial and biological factors. Understanding the cellular mechanisms of adolescents’ sleep onset may provide valuable targets for early detection and prevention of disorders involving prolonged (i.e., homeostatic) or delayed (i.e., circadian) SOT, such as insomnia disorder, delayed sleep phase disorder and their comorbidity. Indeed, inadequate sleep is highly prevalent in adolescents and is associated with multiple medical and psychiatric disorders [4–8]. In addition, inadequate adolescent sleep is a predictor of future sleep disorders in adulthood [9–11].

Several genome-wide association studies (GWAS) in adults have identified single-nucleotide polymorphisms (SNPs) in genes associated with sleep and circadian disorders [12–16]. These GWAS have included metabolic, cell-cycle, synaptic, and circadian-related (i.e., clock) genes. Epigenetic factors may explain the difference between the different SNPs identified across these GWAS investigations. Circadian rhythms are generated through the cyclical expression of specific genes [17] and are heavily regulated by environmental factors, such as the light–dark cycle, diet, heat/cold, among others [18]. DNA methylation (DNAm), the addition of a methyl group to the 5-carbon of cytosine in a Cytosine-phosphate-Guanine (CpG) dinucleotide, is one of the mechanisms by which environmental factors interact with gene expression [19, 20]. DNAm changes are promising biomarkers for developing diagnostic and therapeutic tools partly because they can be seen early in disease processes and the lifespan [21, 22]. Previous studies have identified epigenetic changes associated with inadequate sleep [23]. For example, aberrant DNAm profiles have been identified in adult obstructive sleep apnea syndrome and circadian rhythm shift-work disorder [24, 25], as well as abnormal methylation patterns of clock genes in neurodegenerative and psychiatric disorders [26–28]. This suggests that the etiology of inadequate sleep may have an epigenetic component.

**Fig. 1:**
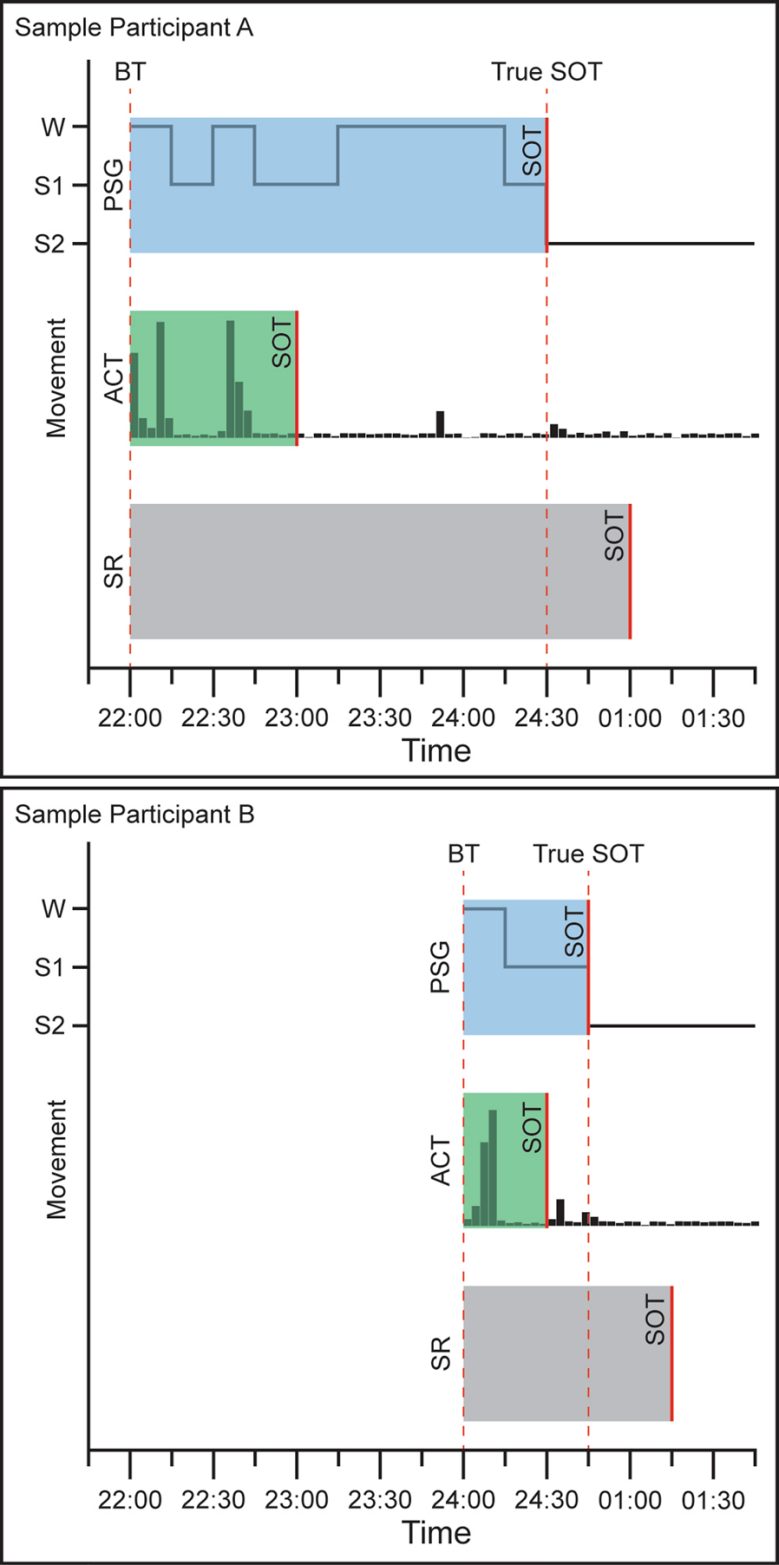
A comparison of sleep initiation measurement methods for two sample participants. Bedtime, sleep onset latency (SOL), and sleep onset time (SOT) are depicted for polysomnography (PSG), actigraphy (ACT), and self-report (SR). PSG SOT is measured as the onset of sleep stage N2, depicted as S2. ACT SOT is calculated from movements and can underestimate sleep latency. SR is measured using participant questionnaires

However, of the prior studies that examined DNAm and sleep in humans, most investigated a small sample of adults with specific sleep disorders [29, 30], and few studies have included sleep measures when studying DNAm in children or adolescents. In children, a meta-analysis found a longitudinal association between actigraphy (ACT)-measured sleep duration and cord blood methylation, but there were no associations for parent-reported sleep measures or cross-sectional associations between DNAm and sleep among children [31]. To our knowledge, three previous studies have investigated DNAm changes associated with sleep phenotypes in adolescence. Huang and colleagues reported an association between subjective sleep duration and DNAm of the circadian-controlled gene, *DOCK1* in 18- to 19-year-olds [32]. Jansen and colleagues identified an association between objective sleep duration and the metabolic genes *PPARA* and *HSD11B2* in 14-year-old girls [33]. Koopman-Verhoeff and colleagues identified an association between ACT-estimated sleep duration and methylation in a module of inter-correlated CpG sites in 10-year-olds [34]. None of these previous studies examined the association between DNAm and sleep initiation phenotypes in adolescents, a key process that deviates during this developmental stage. Additionally, few studies measure objective and subjective sleep outcomes within the same individuals, which may not provide the same results; for example, subjective SOL, as per self-reports, and objective SOL, as per ACT or PSG, assess equally relevant but different dimensions of the same psychobiological process. Self-reports of sleep do not always match or align with objective (i.e., ACT or PSG) measures of sleep [35], as sleep is both a neurobiologically-controlled physiologic need and an observed and modifiable behavior that may be perceived or experienced differently under similar physiologic conditions, as depicted in Fig. 1.

To fill the gap in understanding the epigenetic underpinnings of sleep initiation in adolescents, we performed two sets of analyses investigating the association of DNAm with subjective (i.e., self-reported) and objective (i.e., ACT-measured and in-lab PSG) bedtime, SOL and SOT in adolescents. First, we analyzed intragenic and surrounding methylation sites measured broadly throughout the genome (i.e., gene-wide DNAm). Second, we analyzed specific genes based on previous sleep-related GWAS investigations (i.e., GWAS-informed DNAm) [12–16]. We hypothesized that a later SOT, beyond that expected for a developmental shift in adolescence, will be associated with DNAm in genes previously identified to be involved in sleep regulation and adverse health outcomes.

## Results

### Characteristics of the sample

The demographic characteristics of the 263 participants with DNAm data are presented in Table 1. The sample had an average age of 17 years [13–23], 55.9% were male, and 23.2% identified as a racial/ethnic minority. Participants self-reported an average bedtime at 23:40, an average SOL of 24.1 min, and an average SOT at 24:06. When using at-home ACT, participants showed an average bedtime at 23:59, SOL of 7.5 min, and a SOT at 24:07. During in-lab PSG, participants had an average bedtime at 22:00, SOL of 25.7 min, and a SOT at 22:26.

**Table 1:** Demographic and phenotype statistics of the entire study sample

	N = 263	Range
Age, years	17.3 (2.1)	13–23
Male, %	55.9	
Non-hispanic white, %	23.2	
BMI, percentile %	64.5 (29.0)	0.7—99.8
*Bedtime*		
Self-report, hh:mm	23:40 (1:30)	20:17 – 28:30
Actigraphy, hh:mm	23:59 (1:20)	20:20 – 27:30
Polysomnography, hh:mm	22:00 (0:12)	21:00 – 23:06
*Sleep latency*		
Self-report, minutes	24.1 (19.6)	5—140
Actigraphy, minutes	7.5 (8.5)	1—54
Polysomnography, minutes	25.7 (21.45)	2—131
*Sleep onset*		
Self-report, hh:mm	24:06 (1:35)	20:32 – 30.28
Actigraphy, hh:mm	24:07 (1:19)	20:27 – 27:22
Polysomnography, hh:mm	22:26 (0:26)	21:04 – 24:14

Data are mean (SD) and percentages for continuous and categorical variables, respectively. BMI = body mass index

### Overall DNAm findings

Among the 166,158 analyzable cytosine-phosphate-guanine (CpG) sites, 122,924 intragenic and surrounding sites were annotated onto 12,685 unique genes, while the remaining intergenic sites were not used for the analysis. The gene-wide analysis resulted in 2789 significant sites in 1818 unique genes across the 9 sleep measurements, and the GWAS-informed analysis resulted in 120 sites in 67 unique genes. These sites were statistically significant with q-values < 0.05 (i.e., p-values adjusted for false discovery rate). Manhattan plots for each analysis are located in Additional file 1: Figure S2, and a summary of the number of significant sites for each sleep measure is in Table 2. Additionally, all significant sites are included in Additional file 2.

**Table 2:** Summary of significant methylation sites for each measure of sleep initiation

Sleep measure	Gene-Wide		GWAS-informed	
	Genes	Gene sites	Genes	Gene sites
*Bedtime (hh:mm)*				
Self-report	0	0	0	0
Actigraphy	0	0	0	0
Polysomnography	991	1301	37	62
*Sleep latency (min)*				
Self-report	374	460	20	31
Actigraphy	362	610	11	18
Polysomnography	277	442	10	10
*Sleep onset (hh:mm)*				
Self-report	1	1	0	0
Actigraphy	0	0	0	0
Polysomnography	161	249	7	7

### Enrichment analyses

Both the hypergeometric and permutation tests suggested enrichment of methylation changes among sleep-related genes for subjective bedtime (P_hypergeometric_ = 0.007, P_permutation_ = 0.04), PSG-measured bedtime (P_hypergeometric_ < 0.001, P_permutation_ = 0.005), and subjective SOT (P _hypergeometric_ < 0.001, P_permutation_ = 0.003). The remaining sleep measurements did not show enrichment of sleep-related genes for either the hypergeometric or permutation tests. The hypergeometric and permutation test p-values are summarized in Additional file 1: Table S1. The gene ontology pathway analysis suggested GO terms were overrepresented in PSG-bedtime and SOL by all three measurements after FDR adjustment. The remaining sleep measures did not show enrichment of any GO terms. The top 10 significant biological process (BP) pathways for each analysis are summarized in Table 3, while the remainder are listed in Additional file 2.

**Table 3:** Gene ontology enrichment. Top 10 significant biological process (BP) pathways for each sleep measure

Sleep Measure	GO	Term	Ontology^1^	N^2^	Mapped^3^	q-value
*Bedtime (hh:mm)*						
PSG	GO:0032502	Developmental process	BP	6469	471	2.52E-11
	GO:0048468	Cell development	BP	2034	192	3.17E-11
	GO:0048856	Anatomical structure development	BP	5948	437	5.27E-11
	GO:0007275	Multicellular organism development	BP	5454	402	7.37E-10
	GO:0007399	Nervous system development	BP	2358	207	1.40E-09
	GO:0048731	System development	BP	4887	366	1.43E-09
	GO:0030182	Neuron differentiation	BP	1357	135	5.01E-09
	GO:0030154	Cell differentiation	BP	4234	323	5.19E-09
	GO:0009653	Anatomical structure morphogenesis	BP	2694	225	7.61E-09
	GO:0048699	Generation of neurons	BP	1501	144	8.65E-09
*Sleep latency (min)*						
Self-report	GO:0006357	Regulation of transcription by RNA polymerase II	BP	2539	86	7.0E-03
	GO:0000122	Negative regulation of transcription by RNA polymerase II	BP	873	40	7.0E-03
	GO:0009653	Anatomical structure morphogenesis	BP	2694	89	7.0E-03
	GO:0048598	Embryonic morphogenesis	BP	569	30	7.0E-03
	GO:0007399	Nervous system development	BP	2358	80	7.4E-03
	GO:0022008	Neurogenesis	BP	1613	60	8.4E-03
	GO:0019219	Regulation of nucleobase-containing compound metabolic process	BP	4006	119	9.9E-03
	GO:0009790	Embryo development	BP	992	42	9.9E-03
	GO:0032502	Developmental process	BP	6469	174	9.9E-03
	GO:0006366	Transcription by RNA polymerase II	BP	2695	87	9.9E-03
ACT	GO:0050794	Regulation of cellular process	BP	11,346	374	1.3E-04
	GO:0007399	Nervous system development	BP	2358	108	2.1E-04
	GO:0030154	Cell differentiation	BP	4234	169	2.1E-04
	GO:0048869	Cellular developmental process	BP	4315	171	2.1E-04
	GO:0007275	Multicellular organism development	BP	5454	202	8.9E-04
	GO:0032502	Developmental process	BP	6469	230	1.7E-03
	GO:0048856	Anatomical structure development	BP	5948	214	1.8E-03
	GO:0022008	Neurogenesis	BP	1613	77	1.8E-03
	GO:0050789	Regulation of biological process	BP	12,294	388	1.9E-03
	GO:0065007	Biological regulation	BP	13,019	406	2.1E-03
PSG	GO:0048856	Anatomical structure development	BP	5948	165	2.5E-03
	GO:0044260	Cellular macromolecule metabolic process	BP	8227	212	2.5E-03
	GO:0050793	Regulation of developmental process	BP	2514	84	4.6E-03
	GO:0006366	Transcription by RNA polymerase II	BP	2695	87	6.5E-03
	GO:0006357	Regulation of transcription by RNA polymerase II	BP	2539	83	6.5E-03
	GO:0009058	Biosynthetic process	BP	6216	165	7.0E-03
	GO:0009888	Tissue development	BP	2016	69	7.0E-03
	GO:1,901,576	Organic substance biosynthetic process	BP	6134	163	7.0E-03
	GO:0032502	Developmental process	BP	6469	170	7.0E-03
	GO:0007275	Multicellular organism development	BP	5454	148	7.5E-03

1. N refers to the total number of genes that map to the gene ontology term

2. Mapped genes refers to the number of genes that map to the gene ontology term and have methylation site associations with q < 0.05

### DNAm sites associated with sleep initiation

The top 10 genes from the gene-wide analysis can be found in Table 4 for each of the nine sleep metrics. The top 10 GWAS-informed genes can be found in Table 5 for each of the nine sleep metrics. Self-reported and ACT-measured bedtimes were not significantly associated with any sites after adjustment for false discovery rate (q values > 0.05). PSG-measured bedtime was associated with DNAm of 1301 sites within 991 unique genes included in the gene-wide analysis and 62 sites within 37 GWAS-informed genes.

**Table 4:** Top 10 significant gene-wide methylation sites for each measure

Sleep Measure	Chr	Position	Gene	Β^1^	(SE)	Q value	Function/disease in previous studies^2^	Location
*Bedtime (hh:mm)*								
PSG	chr2	113,240,584	*TTL*	3.8	0.5	9.4E-08	Neoplasms	Intragenic
	chr5	92,956,911	*FAM172A*	2.5	0.3	9.4E-08	Major Depressive Disorder	Intragenic
	chr9	132,258,570	*LINC00963*	1.1	0.1	2.6E-07	Body Height	Intragenic
	chr2	27,434,990	*SLC5A6*	15.4	2.1	3.4E-07	Metabolic Homeostasis	Intragenic
	chr21	37,432,401	*SETD4*	2.3	0.3	3.4E-07	Neoplasms	Intragenic
	chr11	46,722,403	*ZNF408*	0.8	0.1	4.6E-07	Exudative vitreoretinopathy	Intragenic
	chr22	24,237,682	*MIF-AS1*	10.8	1.5	4.6E-07	Autoimmune Disorders	Intragenic
	chr15	68,569,779	*FEM1B*	1.4	0.2	4.6E-07	Diabetes Mellitus	Upstream
	chr3	196,014,497	*PCYT1A*	1.0	0.1	4.6E-07	Spondylometaphyseal Dysplasia	Intragenic
	chr11	64,084,832	*TRMT112*	5.9	0.8	4.6E-07	Eosinophil count	Intragenic
*Sleep latency (min)*								
Self-report	chr10	135,044,054	*UTF1*	− 118	15.0	1.6E-07	Neoplasms	Intragenic
	chr2	209,131,065	*PIKFYVE*	− 2512	335.0	2.2E-07	Fleck corneal dystrophy	Intragenic
	chr14	101,034,180	*BEGAIN*	− 113	15.1	2.2E-07	Synapse structural protein, platelet count	Intragenic
	chr11	101,918,111	*CFAP300*	− 120	16.4	3.2E-07	Ciliary Dyskinesia	Upstream
	chr20	52,000,535	*TSHZ2*	108	14.9	3.2E-07	Waist-Hip Ratio, Blood Pressure	Intragenic
	chr19	5,139,393	*KDM4B*	− 120	16.4	3.2E-07	Medulloblastoma	Intragenic
	chr3	46,008,935	*FYCO1*	110	15.3	5.4E-07	Cataracts	Intragenic
	chr2	74,719,532	*TTC31*	113	15.8	5.4E-07	ADHD, substance abuse	Intragenic
	chr16	29,918,111	*SMG1P2*	100	13.8	5.4E-07	pseudogene	Intragenic
	chr4	1,195,805	*SPON2*	− 101	14.3	7.2E-07	Neoplasms	Intragenic
ACT	chr2	178,937,545	*PDE11A*	− 244	37.4	1.2E-04	Adrenocortical Disease, Depression	Intragenic
	chr1	3,688,710	*CCDC27*	− 758	116.8	1.2E-04	Hemoglobin Level	Downstream
	chr2	200,820,452	*TYW5*	− 424	65.8	1.2E-04	Mathematical ability	Intragenic
	chr3	14,988,783	*FGD5-AS1*	− 445	69.0	1.2E-04	Systolic Blood Pressure	Intragenic
	chr12	862,461	*WNK1*	− 516	81.2	1.4E-04	Neuropathy, Hypertension	Intragenic
	chr11	66,026,044	*KLC2*	− 430	67.7	1.4E-04	Spastic paraplegia, optic atrophy, and neuropathy	Intragenic
	chr18	29,523,469	*TRAPPC8*	− 705	114.4	1.5E-04	Neoplasms	Downstream
	chr19	54,693,401	*MBOAT7*	− 501	81.1	1.5E-04	Intellectual disability	Intragenic
	chr1	1,342,991	*MRPL20*	− 665	106.9	1.5E-04	Body Height	Downstream
	chr8	11,660,746	*FDFT1*	− 468	76.2	1.5E-04	Intellectual Disability, Substance Abuse	Intragenic
PSG	chr12	96,429,291	*LTA4H*	− 1085	154.1	9.3E-06	Atherosclerosis, Depressive Disorder	Intragenic
	chr13	22,032,682	*ZDHHC20*	− 1134	162.2	9.3E-06	Neoplasms	Intragenic
	chr7	75,623,771	*TMEM120A*	− 468	71.3	5.4E-05	Coffee consumption	Intragenic
	chr13	53,024,718	*VPS36*	− 1001	154.6	5.4E-05	Decompression Sickness, Neoplasms	Intragenic
	chr13	111,806,083	*ARHGEF7*	− 1221	195.0	1.1E-04	Psychotic Disorders, Neoplasms	Intragenic
	chr5	70,883,001	*MCCC2*	− 1687	277.7	1.8E-04	3-methylcrotonyl-CoA carboxylase 2 deficiency	Upstream
	chr17	77,770,886	*CBX8*	− 2254	376.9	1.8E-04	Breast Carcinoma	Intragenic
	chr6	150,185,629	*RAET1E-AS1*	− 1565	259.2	1.8E-04	Unknown	Intragenic
	chr1	228,871,178	*RHOU*	− 359	59.7	1.8E-04	Neoplasms	Intragenic
	chr5	177,631,277	*HNRNPAB*	− 1184	198.3	1.8E-04	Neoplasms	Upstream
*Sleep onset (hh:mm)*								
Self-report	chr3	195,447,691	*MUC20*	5	0.8	3.7E-02	Neoplasms	Upstream
PSG	chr7	75,623,771	*TMEM120A*	− 8	1.4	5.8E-03	Coffee consumption	Intragenic
	chr12	96,429,291	*LTA4H*	− 19	3.3	6.0E-03	Atherosclerosis, Depressive Disorder	Intragenic
	chr13	22,032,682	*ZDHHC20*	− 19	3.4	6.0E-03	Neoplasms	Intragenic
	chr12	72,057,164	*ZFC3H1*	− 8	1.5	6.1E-03	Juvenile Arthritis	Intragenic
	chr6	150,185,629	*RAET1E-AS1*	− 27	5.1	8.9E-03	Unknown	Intragenic
	chr6	84,418,880	*SNAP91*	− 8	1.5	9.8E-03	Stroke	Intragenic
	chr22	50,913,327	*SBF1*	− 15	3.0	9.8E-03	Charcot-Marie-Tooth disease	Intragenic
	chr3	14,988,785	*FGD5-AS1*	− 41	8.1	9.8E-03	Systolic Blood Pressure	Intragenic
	chr1	228,871,178	*RHOU*	− 6	1.2	9.8E-03	Neoplasms	Intragenic
	chr22	17,639,892	*HDHD5*	− 8	1.7	9.8E-03	Body Height	Intragenic

1. The units of beta are minutes of sleep latency per 100% change in DNA methylation for SOL and hours per 100% change in methylation for bedtime and SOT. Additional file 2 contains the range, mean, and standard deviation of methylation for each site

2.Gene disease associations were obtained from DisGenet (when available) or Genecards. Associations with stronger evidence scores were prioritized

**Table 5:** Top 10 significant GWAS-informed gene methylation sites

Sleep Measure	Chr	Position	Gene	Β^1^	(SE)	Q value	Function/Disease in Previous Studies^2^	Location
*Bedtime (hh:mm)*								
PSG	chr5	92,956,911	*FAM172A*	2.5	0.3	6.2E-09	Major Depressive Disorder	Intragenic
	chr7	113,727,472	*FOXP2*	1.3	0.2	1.6E-07	Developmental Apraxia	Intragenic
	chr7	1,959,919	*MAD1L1*	2.2	0.3	3.8E-06	Smoking Status, Depression	Intragenic
	chr17	26,926,079	*RSKR*	1.0	0.2	5.9E-06	Reticulocyte count	Intragenic
	chr11	61,583,318	*FADS1*	0.9	0.1	5.9E-06	Neoplasms, Hypertension	Intragenic
	chr11	113,931,707	*ZBTB16*	3.1	0.5	5.9E-06	Skeletal Defects, Depression	Intragenic
	chr5	176,882,353	*PRR7*	3.8	0.7	2.2E-05	NMDA receptor-mediated excitotoxicity	Intragenic
	chr18	56,807,389	*SEC11C*	1.1	0.2	3.6E-05	Neoplasms	Intragenic
	chr20	62,669,859	*C20orf204*	1.1	0.2	3.7E-05	Neoplasms	Intragenic
	chr16	12,541,889	*SNX29*	− 0.6	0.1	1.4E-04	Intelligence	Intragenic
*Sleep latency (min)*								
Self-report	chr14	101,034,180	*BEGAIN*	− 113	15.1	4.8E-08	Synapse structural protein, platelet count	Intragenic
	chr19	5,139,393	*KDM4B*	− 120	16.4	6.1E-08	Medulloblastoma	Intragenic
	chr11	66,384,606	*RBM14-RBM4*	− 154	23.9	1.4E-06	Neoplasms	Intragenic
	chr7	4,212,386	*SDK1*	103	16.0	1.6E-06	Anxiety Disorder	Intragenic
	chr16	23,079,460	*USP31*	108	16.9	1.6E-06	Bone mineral density, chronotype	Intragenic
	chr18	31,803,203	*NOL4*	− 83	14.0	8.1E-06	Circadian Rhythms, Narcolepsy	Intragenic
	chr19	2,595,453	*GNG7*	70	11.9	1.2E-05	Neoplasms	Intragenic
	chr2	236,963,701	*AGAP1*	57	10.3	4.2E-05	Autism	Intragenic
	chr11	122,103,506	*MIR100HG*	86	15.8	5.7E-05	BMI, Intellectual ability	Intragenic
	chr4	148,710,543	*ARHGAP10*	124	26.3	1.5E-03	Atrial Fibrillation	Intragenic
ACT	chr2	178,937,545	*PDE11A*	− 244	37.4	1.1E-05	Adrenocortical Disease, Depression	Intragenic
	chr16	12,070,441	*SNX29*	− 481	77.9	2.4E-05	Intelligence	Upstream
	chr16	56,225,673	*GNAO1*	− 235	43.7	4.7E-04	Seizure Disorders	Intragenic
	chr11	13,298,902	*ARNTL*	− 242	46.2	6.4E-04	Bipolar Disorder	Upstream
	chr5	58,335,105	*PDE4D*	− 700	136.9	7.0E-04	Mental Depression	Intragenic
	chr11	33,757,983	*CD59*	− 432	85.9	7.7E-04	Complement Inhibitor	Intragenic
	chr6	13,201,100	*PHACTR1*	− 52	11.3	2.8E-03	Epilepsy, Coronary Artery Disease	Intragenic
	chr4	83,295,543	*HNRNPD*	− 463	104.5	5.4E-03	Neoplasms	Downstream
	chr5	80,256,552	*RASGRF2*	− 75	17.2	8.5E-03	Neoplasms	Intragenic
	chr10	72,989,362	*UNC5B*	19	4.6	2.7E-02	Subarachnoid Hemorrhage	Intragenic
PSG	chr4	114,682,603	*CAMK2D*	− 728	133.4	7.2E-04	Atrial Fibrillation	Intragenic
	chr11	33,757,983	*CD59*	− 1293	240.6	7.2E-04	Complement Inhibitor	Intragenic
	chr17	17,399,610	*RASD1*	− 995	189.1	7.2E-04	Neoplasms, Circadian Rhythms	Intragenic
	chr12	6,875,771	*MLF2*	− 651	129.0	1.6E-03	Colorectal Carcinoma	Intragenic
	chr17	2,240,898	*SGSM2*	− 7783	1574.7	1.7E-03	Insulin Measurement	Intragenic
	chr5	176,882,488	*PRR7*	− 1812	367.0	1.7E-03	NMDA receptor-mediated excitotoxicity	Intragenic
	chr7	21,582,642	*DNAH11*	− 2132	438.2	2.0E-03	Ciliary Dyskinesia	Upstream
	chr11	61,583,308	*FADS1*	− 834	196.3	2.0E-02	Neoplasms, Hypertension	Intragenic
	chr3	47,018,027	*CCDC12*	− 1551	367.1	2.0E-02	Leukocyte count, Waist-Hip Ratio	Intragenic
	chr15	66,691,039	*MAP2K1*	180	45.3	4.6E-02	Cardio-facio-cutaneous syndrome, MDD	Intragenic

1. The units of beta are minutes of sleep latency per 100% change in DNA methylation for SOL and hours per 100% change in methylation for bedtime and SOT. Additional file 2 contains the range, mean, and standard deviation of methylation for each site

2. Gene disease associations were obtained from DisGenet (when available) or Genecards. Associations with stronger evidence scores were prioritized

Self-reported SOL was associated with differential DNAm of 460 sites in 374 genes included in the gene-wide analysis and 31 sites within 20 GWAS-informed genes.

ACT-measured SOL was associated with DNAm of 610 sites within 362 genes included in the gene-wide analysis and 18 sites within 11 genes included in the GWAS-informed analysis. Of these genes, 19 from the gene-wide analysis and one (*RASGRF2*) from the GWAS-informed analysis were also associated with self-reported SOL.

PSG-measured SOL was associated with 442 sites within 277 genes from the gene-wide analysis and 10 sites within 10 genes from the GWAS-informed analysis. Of these genes, 12 from the gene-wide analysis and none from the GWAS-informed analysis were also associated with self-reported SOL. Furthermore, of the PSG-measured SOL-associated genes, 31 from the gene-wide analysis and 1 (*CD59*) from the GWAS-informed analysis were also associated with ACT-measured SOL. One gene (*SMG1P2*) from the genome-wide analysis was associated with SOL measured by all three methods.

Self-reported SOT was associated with methylation levels in the promotor region of a single gene (*MUC20*) after adjusting for false discovery rate, while ACT-measured SOT was not significantly associated with any sites after adjustment for false discovery rate (q values > 0.05). PSG-measured SOT was associated with 249 sites in 161 genes from the gene-wide analysis and 7 sites within 7 genes from the GWAS-informed analysis. There was a significant overlap between the significant genes associated with PSG-measured SOT and SOL, with 157 genes associated with both.

## Discussion

In a population-based sample of adolescents, we detected changes in leukocyte methylation levels associated with markers of sleep initiation within several genes, including some previously identified in GWAS studies of sleep-related phenotypes in adults. Our data provide evidence for a potential epigenetic link between a delayed sleep onset in youth, measured subjectively or objectively, with specific genes involved in circadian regulation (i.e., *RASD1*, *RAI1*), cardiometabolic disorders (i.e., *FADS1*, *WNK1*, *SLC5A6*), and neuropsychiatric disorders (i.e., *PRR7*, *SDK1*, *FAM172A*). Adolescence is a period of maturational changes in the circadian and sleep homeostatic systems, which can lead to a shift towards later sleep onset. Thus, understanding the cellular mechanisms and sequelae when these changes deviate from what is developmentally expected and result in prolonged or delayed sleep initiation may provide valuable targets for early detection and prevention of comorbid disorders.

**Fig. 2:**
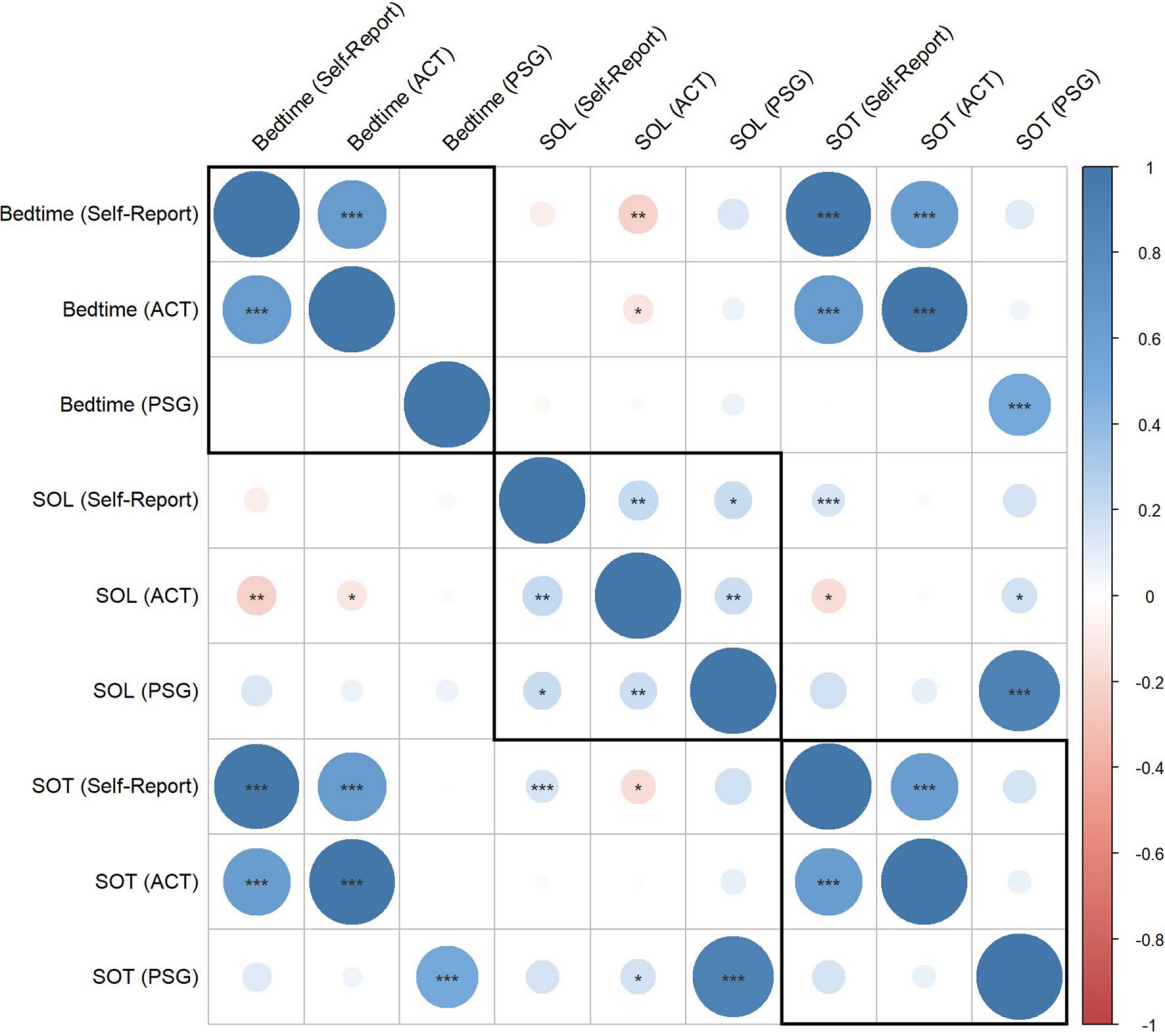
Correlation matrix among sleep initiation measures. The correlation matrix displays the relationships among the different measures of sleep initiation. These measures include bedtime, SOL, and SOT measured by self-report, ACT, and PSG. The color and size of each circle represent the correlation coefficient. The p-value for each correlation is indicated in the matrix: *p < 0.05, **p < 0.01,***p < 0.001

Many of the genes identified in the present study were associated with either bedtime or SOL, but not both. while these measures are related, we expect the gene associations with each to be different, as conceptually depicted in Fig. 1. For example, if two individuals report a late bedtime (1:00 am) but have different SOL (10 vs. 60 min) and only SOL shows significant DNAm for a specific gene, it suggests that the circadian timing of sleep may not have a strong epigenetic association if there is physiologic sleep ability (10 min sleep latency). In contrast, difficulty falling asleep (60 min sleep latency) does have a significant epigenetic association with that site. We also found differential associations with subjective versus objective measures of sleep initiation, which aligns with prior research showing that subjective and objective sleep measures do not always align (Fig. 2) and capture different aspects of sleep behavior and physiology [35].

Interestingly, we did not find significant sites for habitual bedtime as measured by self-report or ACT, but there was signal for bedtime in the lab for the PSG study. In-lab bedtime was standardized with a range from 21:00 to 23:00 for participants to select from; thus, the significant gene sites identified may be likely a result of individual preference within the allowed timeframe that was provided. Although in-lab sleep initiation does not correlate well with at-home sleep initiation (Fig. 2), in-lab PSG is a test for the participants’ physiologic ability to initiate sleep under controlled environmental conditions and may have served to test the function of the de-arousal processes needed to fall asleep.

### Circadian regulation

Methylation changes in sleep-related genes identified in GWAS studies were found to be significantly enriched in the hypergeometric and permutation analysis for bedtime (subjectively and PSG-measured) and subjective SOT. No enrichment of sleep-related genes was observed for the remaining sleep initiation measurements. This may be due to the fact that measuring methylation in peripheral blood did not allow us to identify methylation changes in *CLOCK* genes that may be occurring at the central nervous system (CNS) level. Despite an overall lack of enrichment of sleep-related genes, we observed that DNAm levels in several GWAS-informed genes were related to sleep initiation, including SOL. These associations may be the result of methylation changes in circadian-related genes, such as *RASD1* and *RAI1*, or may be a peripheral consequence of prolonged or delayed sleep initiation. Previously, mutations in *RASD1* mutations have been associated with self-reported circadian preference [36], and mutations in *RAI1*, an important gene involved in circadian melatonin secretion, have been shown to cause abnormal chronology of the sleep–wake cycle and sleep maintenance disorders [37]. In our study population, decreased methylation at different sites within *RAI1* and *RASD1* was significantly associated with a later bedtime, a longer SOL and a later SOT, as assessed by objective measures. Thus, our DNAm findings further support that sleep initiation in adolescents is tightly linked to genes involved in the regulation of circadian rhythms and, that, later sleep initiation may lead to hypomethylation of specific genes.

### Cardiometabolic disorders

Metabolic processes, including glucose and lipid metabolism as well as insulin secretion, are tightly regulated by the circadian system [38]. Sleep disorders have been liked to an increased risk of metabolic-related disorders such as obesity, insulin resistance, hypertension, and type 2 diabetes [39–41]. These associations may be mediated by changes in methylation levels of genes involved in metabolic homeostasis (*SLC5A6*), blood pressure (*WNK1*), and lipid metabolism (*FADS1*). We found that increased methylation at one site within *SLC5A6* was significantly associated with a later PSG-measured bedtime, while decreased methylation at a nearby site was associated with a longer ACT-measured SOL. *SLC5A6*, a gene involved in biotin and pantothenic acid uptake, has been shown to have decreased expression in mice after circadian disruption [42]. We found that decreased methylation in *WNK1*, a gene regulated by the circadian protein Per1 and involved in blood pressure regulation [43], was associated with longer ACT-measured SOL. We found that increased methylation of *FADS1* was associated with a later PSG-measured bedtime, while decreased methylation at a nearby site was associated with a longer PSG-measured SOL. Increased methylation in *FADS1* has been associated with decreased *FADS1* expression and an altered fatty acid profile [44]. These findings further support the idea that prolonged sleep initiation in adolescents may be linked to cardiometabolic health at the epigenetic level.

### Neuropsychiatric disorders

The relationship between inadequate sleep and adverse neuropsychiatric outcomes is well-established, as sleep and circadian disturbances are transdiagnostic across neurodevelopmental, psychopathological, and neurocognitive disorders [45]. Sleep disturbances are common in mood disorders, such as major depressive disorder (MDD), and are consistently associated with psychosis as well as most neurodegenerative disorders, including Alzheimer’s disease (AD). Although methylation at some CpG sites does not necessarily reflect changes in brain tissue for many sites [46], the GO pathway analysis showed an overall overrepresentation of methylation changes in genes involved in neural development with longer habitual SOL measured subjectively and objectively. Additionally, we identified significant methylation sites in individual genes that have been previously linked to neuropsychiatric disorders, including depressive disorders (*FAM172A*), internalizing disorders (*SDK1*), and neurodegenerative disorders (*PRR7*).

We found that increased DNAm in *FAM172A* was significantly associated with a later bedtime, as preferred by subjects for the PSG study. *FAM172A* is a tumor suppressor that has been associated with MDD [47], and maternal circadian disruption has been associated with placental *FAM172A* methylation levels [48]. Previous studies have shown a link between a later bedtime preference and MDD [49], and one potential mechanism for this link could involve methylation. We also found that increased methylation in *SDK1* was significantly associated with a longer SOL, as assessed subjectively but not objectively. *SDK1* mutations have been associated with anxiety disorders [50], and blood methylation levels in *SDK1* have been associated with different forms of psychopathology [51, 52]. Individuals who suffer from anxiety may report taking a long time to fall asleep because anxiety may continue beyond physiologic sleep onset occurs and prolongs the experience of being awake in bed. Our adolescent data further suggests an epigenetic link between difficulty initiating sleep, as measured by subjective reports of SOL, and mental health disorders. Additionally, decreased methylation of *PRR7*, a gene associated with higher-order cognitive functions [53] and linked to neural cell death in neurodegenerative disorders [54], was found to be associated with longer SOL, as objectively assessed by PSG and ACT. These findings provide initial evidence that difficulty initiating sleep during adolescence may contribute to neurocognitive disorders through epigenetic mechanisms.

### Strengths and limitations

Compared to previous studies exploring the relationship between DNAm and measures of sleep, several strengths of our current study may be noted. Our study's use of multiple measures of sleep initiation, including bedtime, SOL, and SOT, allows for a more comprehensive understanding of the relationship between sleep initiation and methylation patterns. Furthermore, we used a combination of both subjective (self-report) and objective (ACT, PSG) measures to better disentangle the multidimensional nature of sleep initiation as both an objectively measurable and perceived phenomenon. We also measured sleep in two distinct contexts: ad-libitum in the home environment (ACT) and under controlled laboratory conditions (PSG). This methodology allowed us to capture a more complete picture of the de-arousal process required for sleep initiation. These relationships were measured in a randomly selected population-based sample of adolescents. Despite these strengths, it is important to acknowledge the limitations of the study. First, there were missing data in the raw methylation sequencing data, which was a result of using a novel whole-genome methylation sequencing technique on the low-yield DNA samples. To maintain the validity of our results, we deliberately excluded those sites with < 10 × coverage or those that were available from < 50% of the sample. In the enrichment analyses, we further limited the scope to CpG sites located within GWAS-informed genes. Second, the sample size (N = 263) of DNA samples was small for genetic studies. Third, DNAm was measured in peripheral blood for a phenotype that is highly centrally regulated. Although some CpG sites have correlation between blood and brain tissue [46], blood DNAm could be a result of environmental or biological factors or outcomes of sleep disturbances or their comorbidities. It is likely that there are epigenetic associations in sleep regulatory regions of the brain that were not detected in our study. Finally, the cross-sectional nature of the analyses does not allow for causal inference. Despite these limitations, our discovery study provides valuable preliminary findings on DNAm and sleep initiation in adolescents.

### Conclusions

In conclusion, we observed in a population-based sample of adolescents that sleep initiation was associated with aggregated changes in DNAm from peripheral blood leukocytes in genes previously associated with circadian and cardiometabolic regulation as well as with neuropsychiatric disorders. We further identified associations between methylation in specific genes and sleep initiation measured subjectively and objectively. However, it should be noted that further validation and replication studies are needed to confirm these findings. Nevertheless, these identified methylation changes may provide valuable targets for early detection and prevention of disorders involving prolonged or delayed sleep initiation, such as insomnia, delayed sleep phase and their comorbidities.

## Methods

### Participants

We analyzed data from 263 adolescents from the Penn State Child Cohort (PSCC). The recruitment and procedures for each study visit have been published elsewhere [55–57]. In brief, 700 children aged 5–12 years were randomly recruited from central Pennsylvania in 2002–2006. Of those, 421 we reexamined in 2010–2013 as adolescents aged 12–23 years (90% of them were younger than 19 years) [58]. No significant differences in demographic characteristics were observed between the 421 reexamined and the 279 lost for the adolescent exam [11, 56]. All participants were evaluated in the Clinical Research Center at Penn State University College of Medicine, including a complete clinical history, physical examination, self-reported questionnaires, a 9-h fixed-time PSG recording in sound-, light-, and temperature-controlled rooms, morning blood draws, and 7-nights at-home ACT monitoring. A total of 263 participants’ morning blood was processed for assaying for DNAm, as described in detail below. Written informed consent from the parent/legal guardian and participants 18 years or older and assent from those younger than 18 were all obtained.

### Sleep initiation measures

Self-reported retrospective questionnaires assessed for bedtime with the questions, *“At what time do you go to bed on weekdays?”* and, *“At what time do you go to bed on weekends?”*. Each subject’s weighted average bedtime was calculated as [(5 * weekdays bedtime in hh:mm + 2 * weekends bedtime in hh:mm)/7 nights]. Self-reported SOL was assessed with the question, “*How long does it take you to fall asleep at bedtime?”* and recorded in minutes. SOT was calculated by adding *weighted average bedtime in hh:mm* + *SOL in minutes, converted to hh:mm*.

An in-lab PSG study was recorded for nine hours in bed from the time of “lights out” (21:00–23:00) until the time of “lights on” (06:00–08:00) to provide a standardized measure of physiologic sleep under controlled in-lab conditions. All PSG recordings were performed on digital electroencephalography, electrooculography, electromyography, electrocardiography and respiratory measures (TWin Recording and Analysis, Grass Telefactor, West Warwick, RI, USA) with a sampling rate of 200 Hz and filter settings at 0.1–70.0 Hz. Independent RPSGTs visually scored all PSG recordings in 30-s epochs following standard criteria and were blind from the participants’ characteristics [59]. In the present study, PSG-identified bedtime was defined as the time of *“lights out,”* ranging from 21:00 to 23:00, based on the participants’ preference within that range. SOL in minutes was defined as the time elapsed from “lights out” to the first epoch scored as stage 2 [59]. SOT was calculated by adding *PSG “lights out” in hh:mm* + *PSG SOL in minutes, converted to hh:mm*.

A 7-night at-home ACT monitoring was performed by participants wearing a tri-axis accelerometer device (GT3X + , Actigraph LLC, Pensacola, FL, USA) for consecutive nights on the wrist of the non-dominant hand. Participants recorded “bedtime” and “rising time” daily in a log, which served to reconcile the bedtimes and rising times objectively recorded by the ACT device. After carefully examining and removing artifacts from the ACT data, all records were scored using Sadeh’s algorithm built in Actilife software (Actigraph LLC, Pensacola, FL, USA). ACT data with fewer than five nights of measurements were excluded. Each subject’s average bedtime was extracted from the seven consecutive nights and recorded in hh:mm. SOL recorded in minutes was also extracted from the automatic scoring in Actilife. SOT was calculated by adding *ACT bedtime in hh:mm* + *ACT SOL in minutes, converted to hh:mm*.

### Genome-wide methylation assay

A total of 391 participants out of the 421 adolescents consented to a blood draw. Fasting peripheral blood samples were collected from each participant and stored at − 80 °C until use. DNA from peripheral blood leukocytes was extracted and subjected to reduced representation bisulfite sequencing (RRBS) using a modified method that has been used in prior studies [60, 61]. Single nucleotide resolution of DNAm in CpG sites and surrounding regions were detected using Illumina HiSeq. 2500. This highly sensitive, multiplexed method generated a specific, reduced representation of the genome of DNA fragments enriched for CpG dinucleotides. Briefly, genomic DNA (minimum 5 ng) was digested with a methylation-insensitive restriction enzyme, MspI, which recognizes CCGG. The digested DNA fragments were purified and subjected to adapter ligation and size selection by AMPure magnetic beads (Beckman Coulter Inc., Brea, CA, USA.). The resulting libraries, covering the target size range between 40 to 200 bp, were quantified by Kapa Library Quantification Kit (Kapa Biosystems Inc., Wilmington, MA, USA). Equimolar libraries were pooled, and unmethylated cytosines (C) were converted to uracils (U) with bisulfite, amplified by polymerase chain reaction, and sequenced. The degree of methylation of each fragment, estimated from the number of converted reads compared to the unconverted reads in each CpG, was calculated. Base calls of bisulfite-treated sequencing reads with phred quality scores < 20 and length < 35 bp were trimmed, and the adaptor was cut using trim_galore V0.3.3 (Babraham Bioinformatics, Cambridge, UK). The resulting reads were mapped to the hg19 assembly, and methylation calls were performed using Bismark v0.10.1 (Babraham Bioinformatics, Cambridge, UK). After alignment, approximately 1.6 million methylation levels were detected at more than 10 × coverage.

### Statistical analyses

To ensure the validity of the DNAm data and statistical inferences of the analyses, we excluded bases with < 10 × coverage or available from < 50% samples, leaving a total of 166,158 analyzable sites. Among the 391 blood samples, 263 yielded an adequate amount of DNA to be sequenced. There was no significant difference in the major demographic characteristics between the subjects whose DNA was sequenced and the original cohort, as previously reported [61]. We used multivariable-adjusted linear regression models to investigate the relationship between sleep initiation measurements and DNAm. In these models for bedtime, SOL and SOT, the respective sleep measurement and site-specific methylation levels were treated as dependent and independent variables, respectively, while adjusting for age, race, sex, BMI percentile, and batch effects. Sites from the converged models were annotated based on the hg19 assembly in R [62]. Gene borders were extended to include − 1500 bp upstream and + 500 bp downstream the gene boundaries. All intragenic and surrounding sites were then taken forward for enrichment analyses. A q value < 0.05 was used to determine the significance of the gene-wide and GWAS-informed analysis using the Benjamini and Hochberg method to adjust for the false discovery rate (FDR) [63]. All analyses were performed by using R [64].

### Enrichment analyses

We identified a list of genes previously determined to be related to sleep and circadian phenotypes in GWAS [13–16]; thus, the intragenic sites were categorized into gene-wide sites and GWAS-informed sites. Gene-set enrichment was assessed with a hypergeometric test and a permutation test with 1000 permutations [65]. These tests compared the distribution of CpG sites, whose methylation level was associated with each sleep measure at p < 0.05 level intragenic to gene-wide and GWAS-informed genes. To test for functional enrichment of significant sites, we performed gene ontology (GO) pathway analysis on genes annotated to CpGs with significant FDR [63] adjusted p-values (i.e., q-values) using the *limma* package in R [66].

### Supplementary Information

**Additional file 1.** **Table S1**: Enrichment Analysis p-values and Supplementary

**Additional file 2.** Significant results for the all-gene and GWAS-informed analyses.Significant Pathway Analysis Results.

## Data Availability

The datasets used and/or analyzed during the current study are available from the corresponding author on reasonable request.
