# Supplementary material for: Objective and subjective measures of sleep initiation are differentially associated with DNA methylation in adolescents
Source: Clin Epigenetics. 2023 Aug 26;15:136. doi: 10.1186/s13148-023-01553-2 (PMC10464279; doi:10.1186/s13148-023-01553-2)
Supplement: Supplementary file 1 — Additional file 1. Table S1: Enrichment Analysis p-values and Supplementary [file 13148_2023_1553_MOESM1_ESM.docx]

**Supplementary Table 1. Enrichment Analysis p-values**

| Sleep measurement | Hypergeometric test p-value | Permutation p-value |
| --- | --- | --- |
| Bedtime (Self-Report) | 0.007* | 0.036* |
| Bedtime (ACT) | 0.5 | 0.76 |
| Bedtime (PSG) | 0.0003* | 0.005* |
| SOL (Self-Report) | 0.18 | 0.36 |
| SOL (ACT) | 0.18 | 0.39 |
| SOL (PSG) | 0.57 | 0.76 |
| SOT (Self-Report) | 0.0001* | 0.003* |
| SOT (ACT) | 0.64 | 0.82 |
| SOT (PSG) | 0.13 | 0.28 |

* Statistically Significant p < 0.05

Figure S1. Manhattan Plots of adjusted P-values


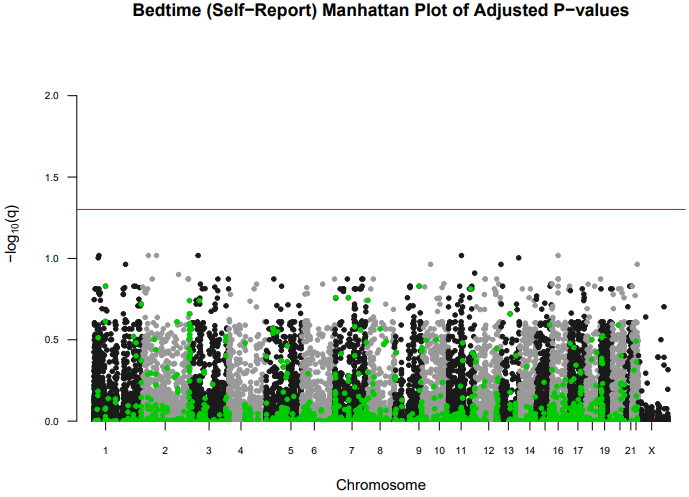

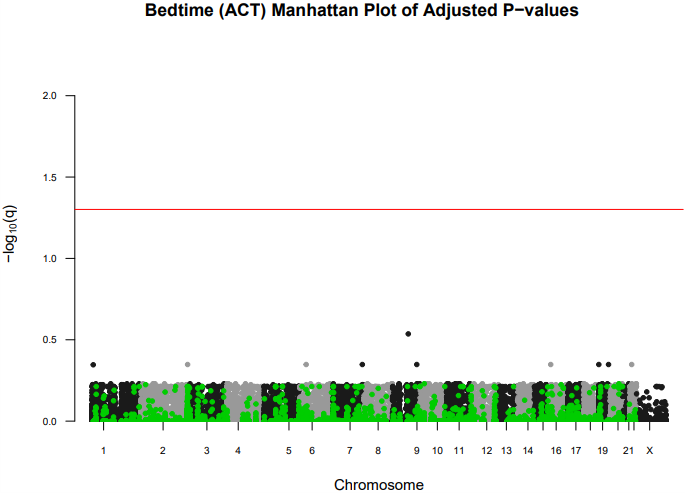

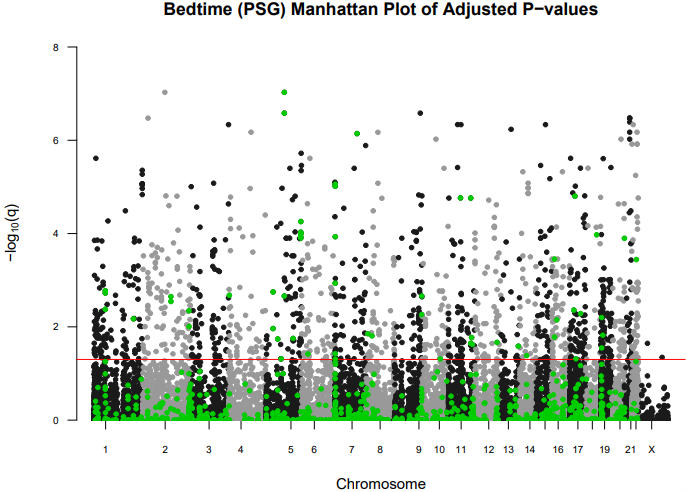


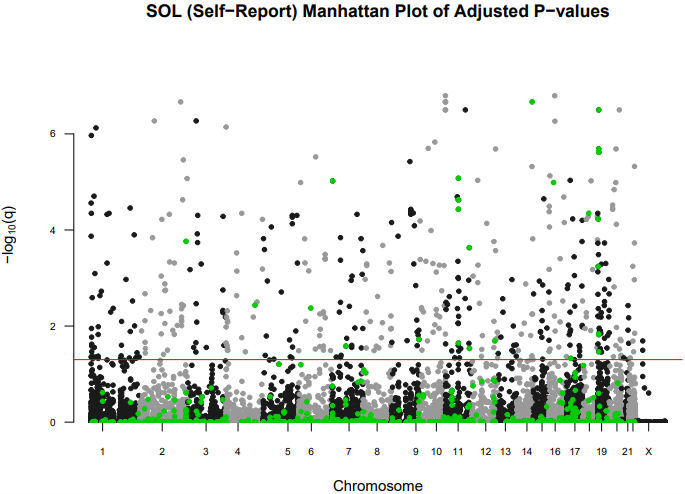

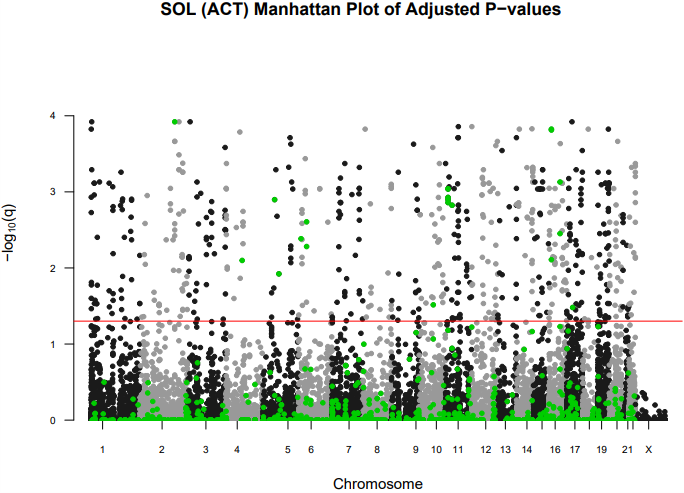

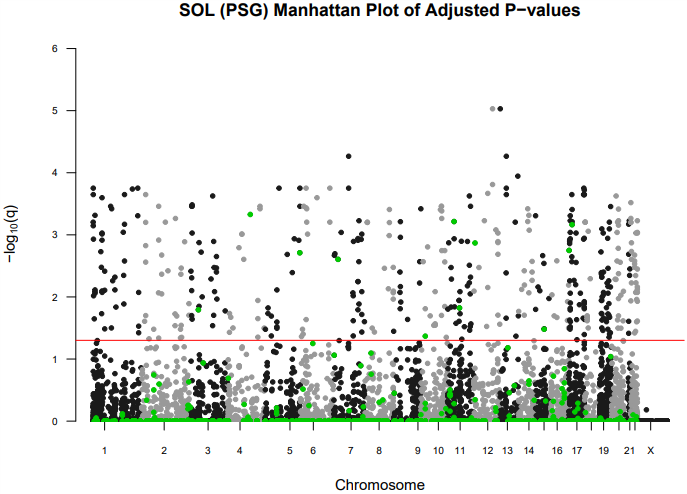


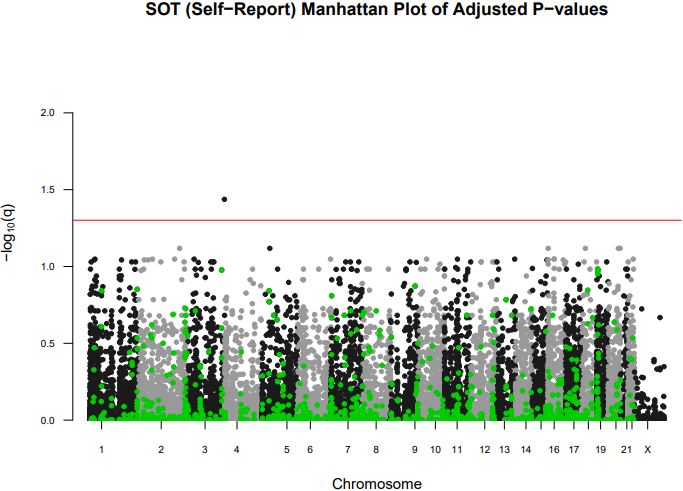

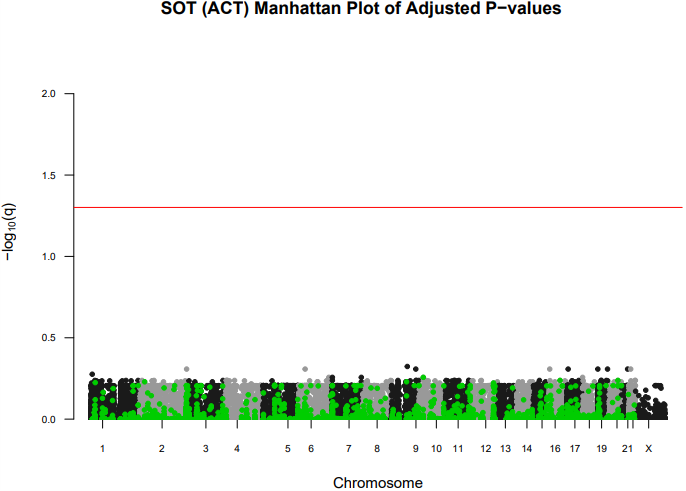

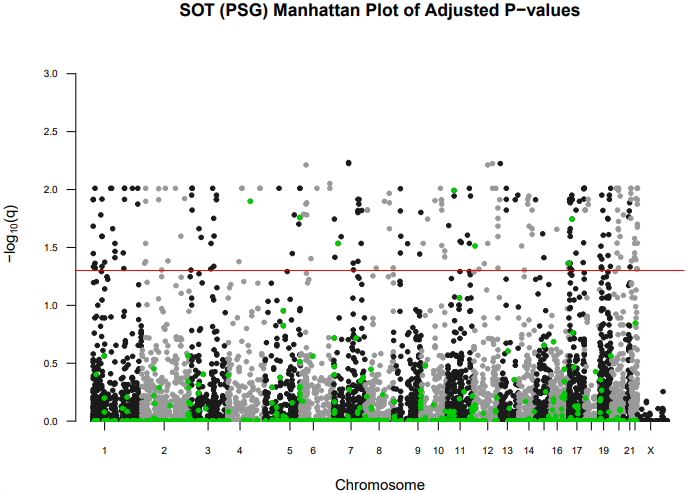


All represented sites fall either within genes or within specific proximities to genes: 1500 base pairs upstream or 500 base pairs downstream. The green dots specifically highlight the methylation sites that are within or near genes identified by previous genome-wide association studies (GWAS), while the remaining sites are associated with other genes. Red lines indicate a threshold where the q-value is less than 0.05. These q-values represent p-values that have been adjusted using the Benjamini and Hochberg method.
